# Predictive modeling of tax compliance risks: A comparative study of machine learning approaches

**DOI:** 10.1371/journal.pone.0331715

**Published:** 2025-09-30

**Authors:** Lan Yang

**Affiliations:** Southeast Bangkok University, Bangkok, Thailand; State University of New York at Oswego, UNITED STATES OF AMERICA

## Abstract

Modern enterprises grapple with complex financial data and multidimensional risk interdependencies in their operations. Machine learning offers transformative potential for tax risk assessment and smart auditing solutions. This research analyzes 3,232 tax records from regional manufacturing and service sectors (2021–2023) to evaluate three predictive models: SVM, XGBoost, and Random Forest. Results demonstrate Random Forest’s superior performance, achieving 92.00% (manufacturing) and 93.39% (service) accuracy – substantially outperforming XGBoost and SVM (85–90%). Key manufacturing risk indicators follow a “high tax-high volatility-high scrutiny” pattern, with tax burden rate (0.129 weight), profit fluctuation (0.100), and audit frequency (0.091) being most predictive. Service sector risks manifest as “volatility-declaration-tax burden” dynamics, where profit volatility (0.142) emerges as the strongest predictor. These findings both validate machine learning’s efficacy in tax analysis and equip regulators with intelligent risk management tools.

## 1. Introduction

With the continuous improvement of tax policies and the advancement of the digitalization process in tax management, the analysis and management of corporate tax-related behavior has become an important issue to ensure tax fairness and economic order [1,2]. However, traditional tax management methods struggle to achieve precise and efficient risk identification and early warning in the face of an increasingly complex economic environment and multidimensional data, leading to increased management costs and limited effectiveness [3,4]. Against this backdrop, exploring corporate tax-related behavior features and identifying potential risks through more systematic analytical methods has become a key direction for improving the quality of tax management [5]. Based on this, the study starts from the characteristics of corporate tax-related behavior and uses machine learning models to explore scientific and systematic risk management ideas, aiming to improve the standardization and effectiveness of tax management.

In existing research on corporate financial anomaly detection and tax-related risk identification, traditional machine learning models and statistical learning methods are widely employed to enhance the scientific rigor and automation level of risk assessment. In terms of machine learning approaches, Xu & Kong (2024) utilized the Random Forest (RF) model to construct a financial compliance classifier, which demonstrated strong robustness and classification performance when handling high-dimensional enterprise feature data [6]. Maina et al. (2023) introduced the XGBoost algorithm into real-time tax fraud detection, significantly improving the model’s recognition accuracy and recall capability in imbalanced data environments [7]. Zhao (2022) proposed an improved Random Forest ensemble model that exhibited superior feature extraction and generalization capabilities in anomaly detection tasks [8]. These studies suggest that models such as RF and XGBoost possess strong identification and prediction abilities in enterprise data environments characterized by complex structures and heterogeneous variables. In terms of statistical learning methods, Pavlicko & Mazanec (2022) employed the Logistic Regression model to analyze the relationship between corporate financial indicators and tax risks [9]. Their findings indicated that static indicators such as the debt-to-asset ratio and profit margin were significant predictors in risk forecasting. Due to its simplicity and strong interpretability, logistic regression is frequently used in the construction of traditional tax compliance models. Although the aforementioned models have achieved initial results in the analysis of tax-related behavior, most studies focus on model construction or the application of techniques in single domains, lacking systematic comparative research on multi-source tax-related data integration, industry heterogeneity handling, and model adaptability. Against the backdrop of increasingly complex corporate tax-related behaviors, a key challenge in this field remains how to construct high-performance and interpretable risk prediction models based on multi-dimensional indicators. The study integrates financial indicators, non-financial features, and time-series data to build a comprehensive tax risk assessment indicator system, as well as a comprehensive analysis framework using SVM, XGBoost, and Random Forest models, to comprehensively improve the accuracy of tax-related behavior identification and the scientific nature of risk management. The research finds that a multi-indicator feature system significantly outperforms single features, showing outstanding performance in capturing corporate tax-related behavior patterns and dynamic changes, with Random Forest performing the best, significantly outperforming other models. In addition, service industry enterprises exhibit more stable model classification performance due to the relatively more uniform distribution of features. This result indicates that the optimization of the multi-indicator feature system and the adaptation of model selection are of great significance for enhancing the scientific nature of intelligent analysis and risk management of corporate tax-related behavior. The research findings provide important references for tax authorities in formulating scientific risk prevention and control strategies, while offering practical guidance for enterprises to optimize tax-related behavior management and reduce compliance costs, making important contributions to both theoretical research and practical innovation in tax risk management.

The structure of this paper is as follows: the first section is the introduction, which introduces the research background, problems, and objectives; the second section is the literature review, which systematically organizes relevant research on the analysis of corporate tax-related behavior and risk management; the third section is the research design, which explains the data sources, indicator selection, model construction, and evaluation methods; the fourth section is the empirical analysis, which demonstrates and analyzes the application effects of machine learning models in tax-related behavior analysis and risk management; the fifth section is the discussion, which compares the research findings with those of other scholars and highlights the unique contributions of this paper; the final section is the conclusion, which summarizes the research findings and proposes directions for future research.

## 2. Literature review

The intelligent analysis and risk management of corporate tax-related behavior is an important field of modern financial technology research. With the acceleration of the digitalization process in tax management, various enterprises face an increasingly complex risk environment in their financial operations, making the efficient integration of multi-dimensional data and the precise identification of risk factors a research hotspot [10]. The introduction of machine learning technology has transformed tasks such as anomaly detection in corporate financial data, tax risk prediction, and compliance auditing from static analysis to dynamic modeling [11,12]. Research based on different models and algorithmic frameworks has gradually expanded, building efficient risk early warning systems and methods for detecting tax-related behavior, which not only provide strong support for financial supervision but also offer intelligent pathways for enterprises to optimize tax compliance strategies.

Financial anomaly detection is an important application of machine learning technology. Researchers have built efficient anomaly identification models to analyze and warn of potential abnormal behavior in corporate financial data. Wang et al. (2022) developed an XGBoost ensemble model to identify abnormal tax burden levels among enterprises, significantly enhancing classification performance under imbalanced sample conditions [13]. Mojahedi et al. (2022) compared the performance of Logistic Regression, Decision Tree, and Support Vector Machine (SVM) on tax-related datasets, finding that Logistic Regression performed stably when dealing with low-dimensional data, while SVM was more suitable for anomaly detection in nonlinear scenarios [14]. Zhang et al. (2023) designed a triple feature selection mechanism based on the Random Forest model, which effectively improved detection accuracy and stability under multi-dimensional data conditions [15]. Liu et al. (2024) further proposed a distribution-transformation-based Random Forest approach, demonstrating strong performance in detecting abnormal behaviors in enterprises’ inter-period financial fluctuations [16]. Meanwhile, George et al. (2024), through empirical research, found that traditional machine learning models still hold significant application value in identifying financial fraud risks, particularly in enterprise scenarios with moderately sized data samples and clearly defined feature variables [17].

In the area of risk prediction and assessment, research focuses on improving models’ ability to recognize and predict multi-dimensional risk factors, such as credit risk and market risk. Suganya et al. (2023) proposed a Random Forest ensemble algorithm based on a weighted voting mechanism, which significantly improved prediction accuracy and recall in financial fraud detection [18]. Mir et al. (2024) employed a Support Vector Machine model optimized with adaptive thresholding, enhancing the identification capability for financial risks in small and medium-sized enterprises [19]. Maheswari et al. (2024) constructed a hybrid feature selection mechanism that combines XGBoost and Random Forest for credit default prediction, improving the model’s capacity to handle high-dimensional heterogeneous variables [20]. In an empirical study on corporate risk management, Zhang (2024) applied Logistic Regression to identify tax-related risk factors and incorporated corporate behavioral data to build a risk scoring system, thereby enhancing both the interpretability and operational applicability of the model [21]. Cernisevs et al. (2023) further integrated traditional classifiers to assess the impact of different compliance behaviors on financial risk, suggesting that in scenarios characterized by complex data sources and clearly classified indicators, traditional models still possess strong generalization capabilities [22].

In compliance and audit risk management, the introduction of machine learning technology has provided intelligent solutions for corporate financial compliance. Sawalha et al. (2023) developed an ensemble classifier that integrates corporate financial ratios and governance variables, demonstrating superior robustness and accuracy in audit data early warning tasks [23]. Vullam et al. (2023) proposed an integrated classification model for tax compliance alerts, applying it to the automatic classification of financial statements from small and medium-sized enterprises, achieving an identification accuracy exceeding 90% [24]. In addition, Diao (2024) employed a hybrid model combining Support Vector Machine and Gradient Boosting Trees in a large-sample enterprise risk analysis, empirically validating its capability to capture features from historical audit data [25]. Phong et al. (2022) constructed an evaluation framework for corporate financial behavior based on the Logistic Regression model, which was used to identify high-risk enterprises with tendencies toward tax violations [26]. Chalevas et al. (2025) utilized the XGBoost model to analyze the relationship between corporate tax avoidance behavior and financing costs, finding that the model demonstrated strong performance in identifying compliance-related features [27].

Traditional machine learning models, with their clear structure, high interpretability, and computational efficiency, have demonstrated strong applicability in tasks such as tax-related behavior identification, risk prediction, and audit supervision. Although existing studies have yielded fruitful results across various application scenarios, there remain notable gaps in the horizontal comparison of model performance, the delineation of applicability boundaries, and the development of model selection mechanisms—particularly in the context of systematic comparative analyses for corporate tax risk prediction.To address these issues, this paper predicts corporate tax-related behavior based on three machine learning models: Support Vector Machine (SVM), XGBoost, and Random Forest, selects the optimal model through performance metrics, and uses the optimal model for more in-depth analysis of tax risk behavior.

## 3. Research design

### 3.1. Data source and preprocessing

The data used in this study were obtained from the tax authority of a specific region under the State Taxation Administration of China and cover tax filing records, audit information, and financial statement data of manufacturing and service enterprises from 2021 to 2023. The original dataset contained a total of 3,010 enterprise samples, including approximately 1,316 from the manufacturing sector and 1,694 from the service sector. Label information was provided by the tax authority based on audit results: enterprises identified as having significant tax violations during this period were labeled as “with tax risk,” while the others were labeled as “without tax risk.” In the original dataset, among the manufacturing enterprises, 214 were labeled as having tax risk and 1,102 as non-risk; among the service enterprises, 393 were identified as tax-risk and 1,301 as non-risk.

To ensure data quality, the following preprocessing steps were undertaken:

(1) Removal of missing and abnormal values: Records with missing core fields (such as tax burden ratio and profit margin) or logical inconsistencies were removed. Abnormal values were identified using the 3σ rule, and any record with an absolute z-score greater than 3 was excluded. This step removed 230 invalid records.
(2) Normalization of variables: All continuous variables (e.g., operating revenue, tax burden ratio, quarterly volatility indicators) were standardized using the z-score normalization method to eliminate differences in units. Non-continuous variables (e.g., industry category) were encoded using one-hot encoding to enhance model interpretability and convergence efficiency.
(3) Training and testing set division: To prevent data leakage, the remaining 2,780 cleaned samples were randomly split into a training set (1,946 samples) and a testing set (834 samples) in a 7:3 ratio. The proportions of manufacturing vs. service enterprises and tax-risk vs. non-risk samples were kept consistent across both subsets.
(4) SMOTE oversampling in training set: Since the cleaned data still exhibited class imbalance with fewer tax-risk samples, the Synthetic Minority Over-sampling Technique (SMOTE) was applied to the training set to oversample the “with tax risk” class. SMOTE used a k-nearest neighbor parameter of 5 to avoid generating samples that deviated excessively from the original distribution. After data cleaning and processing, a total of 2,389 valid training samples were retained, including 1,123 from the manufacturing sector (556 with tax risk and 567 without tax risk) and 1,266 from the service sector (629 with tax risk and 637 without tax risk). This treatment significantly mitigated the class imbalance issue and enhanced the model’s capacity for risk identification. The testing set retained its original distribution to evaluate the model’s generalization performance under realistic business scenarios.

### 3.2. Indicator selection

The initial dataset included 32 potential feature variables spanning multiple dimensions, including financial information, basic enterprise attributes, tax-related behavior data, and time-series characteristics. During the feature selection process, variables were evaluated based on data completeness (e.g., proportion of missing values), preliminary correlation with the risk label (e.g., information gain, correlation coefficients), and business interpretability. Variables that were highly redundant, severely imbalanced in distribution, or demonstrated weak discriminatory power were removed. As a result, 14 key indicators across financial, non-financial, behavioral, and time-series dimensions were initially selected as modeling features. To further verify the importance and ranking stability of these indicators within the models, the study introduced the Recursive Feature Elimination (RFE) method as a supplementary analytical tool. The RFE results showed that the majority of the 14 selected indicators consistently ranked among the top features across different models, confirming their representativeness and effectiveness in risk identification. The indicator system is shown in Table 1. Financial and behavioral characteristic indicators provide a static description of corporate tax risk, while time series characteristics and dynamic behavior indicators capture changes over time, offering richer feature information for the machine learning models.

**Table 1:** Corporate tax-related behavior analysis indicator system.

Indicator Category	Name	Definition
Financial Indicators	Operating Revenue (AR)	Annual total revenue of the enterprise (in 10,000 RMB)
	Profit Margin (PM)	Net profit/ Operating revenue (%)
	Tax Burden Ratio (TBR)	Actual tax paid/ Operating revenue (%)
	Current Ratio (CR)	Current assets/ Current liabilities (%)
	Debt-to-Asset Ratio (DAR)	Total liabilities/ Total assets (%)
Behavioral Characteristics	Filing Frequency (FF)	Number of tax filings per year (times/year)
	Tax Audit Frequency (TIF)	Cumulative number of tax audits for the enterprise
	Audit Issue Amount (IIA)	Amount of issues identified in tax audits (in 10,000 RMB)
Time Series Characteristics	Quarterly Tax Burden Volatility (QTB)	Standard deviation of tax burden changes per quarter
	Quarterly Profit Volatility (QPV)	Standard deviation of profit changes per quarter
	Revenue Growth Rate (RGR)	(Current period revenue – Previous period revenue)/ Previous period revenue (%)(%)
Non-Financial Indicators	Registered Capital (RC)	Total registered capital of the enterprise (in 10,000 RMB)
	Number of Employees (NE)	Total number of employees in the enterprise (persons)
	Years Since Establishment (FA)	Current year – Registration year (years)

### 3.3. Machine learning model construction

In order to achieve precise analysis and risk management of corporate tax-related behavior, this study selected three classic machine learning models: Support Vector Machine (SVM), Gradient Boosting Decision Trees (XGBoost), and Random Forest. These three models demonstrate strong performance in handling small- to medium-sized datasets, high-dimensional features, and nonlinear relationships, and they have been widely applied in financial compliance analysis and risk identification due to their stability and interpretability [28,29]. Compared with deep learning models such as Long Short-Term Memory (LSTM), although LSTM exhibits certain advantages in processing complex time-series data [30], the time-series variables in this study have already been structurally extracted using statistical measures such as volatility. As a result, the models require less dependence on long sequence information. Employing LSTM would not only yield limited performance improvement but may also reduce model interpretability due to its higher complexity. In addition, ensemble methods such as Voting and Stacking were also tested during the modeling process. However, due to limitations in sample size and the generalization ability of individual base learners, these methods exhibited overfitting issues during cross-validation and were therefore not included in the final modeling framework. Taking into account prediction performance, interpretability, controllability, and the tax authorities’ practical demand for result transparency, this study adopts Support Vector Machine (SVM), XGBoost, and Random Forest as the primary models for comparative analysis. These models not only meet the requirements of tax-related risk classification tasks for nonlinear modeling but also support feature importance analysis, which helps in identifying key risk factors.

1. SVM model construction

Support Vector Machine (SVM) is a classification model based on the principle of structural risk minimization. It achieves classification by constructing a hyperplane that maximizes the margin between class boundaries. The optimization objective can be expressed as follows:


$$\begin{array}{c} \min_{𝐰,b,\xi }\;\frac{\text{1}}{\text{2}}|𝐰{|}^{2}+C\sum_{i=1}^{n}\xi_{i}\;s.t.\;y_{i}({𝐰}^{T}\phi (x_{i})+b)\geq 1-\xi_{i},\;\xi_{i}\geq 0 \end{array}$$ (1)

where, ϕ(x) denotes the kernel function mapping, C is the penalty parameter, and ξᵢ represents the slack variable. The model adopts the Radial Basis Function (RBF) kernel, and a grid search was conducted to optimize C ∈ {0.1, 1, 10} and *γ* ∈ {0.01, 0.1, 1}. Based on cross-validation, the optimal parameter combination was determined to be C = 1 and *γ* = 0.1. A relatively low *γ* value was selected to control the complexity of the kernel function and mitigate the model’s tendency to overfit the training data.

2. XGBoost model construction

XGBoost is an ensemble learning algorithm based on the Gradient Boosting Decision Tree (GBDT) framework. Its objective function consists of a training loss component and a structural regularization term, which can be expressed as follows:


$$L(t)=\sum_{i=1}^{n}l(y_{i},\hat{y}i^{\left(t\right)})+\sum k=1^{t}\Omega (f_{k}),\;\Omega (f)=\gamma T+\frac{\text{1}}{\text{2}}\lambda \sum_{j=1}^{T}w_{j}^{2}$$ (2)

where, l represents the loss function, Ω(f) denotes the regularization term, T refers to the number of leaf nodes, and wⱼ is the score assigned to the j-th leaf node. The model was optimized using a randomized search within the following parameter space: learning_rate ∈ {0.01, 0.05, 0.1}, max_depth ∈ {3, 5, 7}, subsample ∈ {0.6, 0.8, 1.0}, and regularization coefficient α ∈ {0, 0.5, 1.0}. An early stopping mechanism with a patience of 10 rounds was also introduced. The optimal parameter combination, determined through five-fold cross-validation, was learning_rate = 0.1, max_depth = 5, subsample = 0.8, and α = 0.5. This configuration achieved the best F1-score performance, balancing model complexity control with prediction accuracy. Notably, the regularization parameter α and the subsample ratio together formed the model’s complexity control mechanism, effectively reducing overfitting risk and improving generalization on the test set.

3. Random forest model construction

Random Forest is an ensemble model based on the Bagging strategy, which enhances model stability and generalization capability by aggregating the voting results of multiple decision trees. A grid search was used to optimize the following hyperparameters: the number of trees (n_estimators ∈ {100, 200, 300}), the maximum tree depth (max_depth ∈ {5, 10, 15}), and the minimum number of samples required to split an internal node (min_samples_split ∈ {2, 5, 10}). The final selected parameters were n_estimators = 200, max_depth = 10, and min_samples_split = 2. Although both XGBoost and Random Forest are tree-based models, their structural differences are fundamental: XGBoost is a serially constructed weighted boosting model, whereas Random Forest is a parallel ensemble model based on independent decision trees with majority voting. Therefore, discrepancies in the optimal max_depth between the two models are expected and do not affect the validity of performance comparisons under the same evaluation metrics. This parameter configuration demonstrated robust performance in cross-validation, with strong capability for risk identification and good model interpretability. Furthermore, Random Forest constructs multiple independent sub-models using the Bagging strategy and evaluates generalization performance through out-of-bag (OOB) error estimation, making it inherently resistant to overfitting.

### 3.4. Evaluation metrics

This study selected five evaluation metrics: accuracy, precision, recall and F1-score to comprehensively assess the performance of SVM, XGBoost, and Random Forest models in the tasks of intelligent analysis of tax-related behavior and risk management.

1. Accuracy

Accuracy is the most commonly used overall performance metric, suitable for datasets with balanced class distributions. It reflects the model’s overall prediction ability and is calculated as follows:


$$Accuracy=\frac{TP+TN}{TP+TN+FP+FN}$$

Where TP is True Positive, meaning the model correctly predicted a positive sample as positive; TN is True Negative, meaning the model correctly predicted a negative sample as negative; FP is False Positive, meaning the model incorrectly predicted a negative sample as positive; FN is False Negative, meaning the model incorrectly predicted a positive sample as negative.

2. Precision

In tax-related behavior analysis, false positives (incorrectly predicting low-risk enterprises as high-risk) may lead to unnecessary tax investigations and other resource waste. Therefore, precision is used to measure the model’s prediction accuracy when identifying risky samples, reducing misjudgments. The formula is:


$$Precision=\frac{TP}{TP+FP}$$

3. Recall

In tax audit tasks, false negatives (failing to identify high-risk enterprises as low-risk) can lead to severe consequences such as tax revenue loss. Therefore, recall is a key focus, ensuring that the model can identify as many high-risk enterprises as possible, reducing the chances of missed reports and improving the comprehensiveness of risk identification. The formula is:


$$Recall=\frac{TP}{TP+FN}$$

4. F1-Score

The F1-score is particularly important for datasets with imbalanced class distributions. When the model’s precision and recall trade-off, the F1-score provides a more comprehensive performance measurement, preventing the model from excelling in one metric while neglecting the other. The formula is:


$$F1=2\times \frac{Precision\times Recall}{Precision+Recall}$$

## 4. Research results and analysis

### 4.1. Descriptive statistics

This paper presents descriptive statistics for all variables, with results shown in Table 2. From the financial indicators, the means of annual revenue and profit margin indicate that the overall business performance of the sample enterprises is stable. However, the large standard deviations reflect significant differences between enterprises, which may be due to the different profit structures of manufacturing and service industries. Some enterprises have tax burdens exceeding 60%, suggesting insufficient tax planning or business strategies that focus on high-tax products. Regarding tax-related behavior characteristics, some enterprises exhibit high frequencies of tax reporting and tax audit occurrences, indicating complex financial activities that make them likely targets for tax authorities. The high values of audit-related amounts further suggest that some enterprises face risks in compliance management and need to improve their financial internal control mechanisms. Time series characteristics show that some enterprises experience significant financial fluctuations between quarters, which may be influenced by market environment changes or seasonal business activities. These fluctuations could be related to tax planning or financial anomalies. Although most enterprises show stable revenue growth, some experience negative growth, indicating market pressure. Non-financial indicators show that the sample enterprises are primarily small and medium-sized businesses, with a relatively high number of years in operation, reflecting the mature nature of the sample enterprises.

**Table 2:** Descriptive statistics results.

Variable	Mean	Std	Min	Max
Operating Revenue (AR)	43.121	17.453	12.155	73.002
Profit Margin (PM)	23.895	7.432	3.419	44.937
Tax Burden Ratio (TBR)	32.184	11.214	1.316	61.605
Current Ratio (CR)	41.019	2.743	18.978	63.737
Debt-to-Asset Ratio (DAR)	26.672	17.978	19.313	34.788
Filing Frequency (FF)	57.328	20.718	16.174	97.567
Tax Audit Frequency (TIF)	47.803	22.868	1.962	95.164
Audit Issue Amount (IIA)	53.159	22.243	13.688	91.586
Quarterly Tax Burden Volatility (QTB)	38.862	15.359	8.809	67.832
Quarterly Profit Volatility (QPV)	48.145	22.731	2.457	93.751
Revenue Growth Rate (RGR)	17.545	4.085	9.909	27.079
Registered Capital (RC)	17.251	6.459	0.697	35.679
Number of Employees (NE)	20.531	1.484	18.187	23.618
Years Since Establishment (FA)	26.421	10.269	5.183	46.026

### 4.2. Machine learning model prediction results

**Fig 1:**
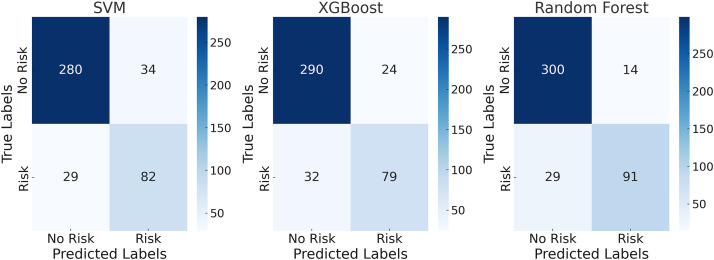
Confusion Matrix for the Manufacturing Test Set with 14 Evaluation Indicators.

Fig 1 presents the classification results of the manufacturing test set using 14 feature indicators. The results show that among the three models, the Random Forest model performed the best, with an accuracy of 92.00%, precision of 86.67%, recall of 81.98%, and an F1 score of 84.26%. This excellent performance can be attributed to the robustness of Random Forest in handling high-dimensional data, which effectively reduces the risk of overfitting by integrating multiple decision trees, thereby capturing data features comprehensively. It is worth noting that the results achieved by the proposed model not only outperform the other models evaluated in this study, but also demonstrate a significant advantage when compared with existing research. For example, Zou (2024) employed a decision tree model for corporate financial compliance analysis, achieving an accuracy of 86.3% [31], while Anusha et al. (2023) constructed a Random Forest model for real-time tax fraud detection, which reached an accuracy of approximately 88.5% on a small-sample financial dataset [32]. In contrast, the Random Forest model in this study maintained an accuracy exceeding 92% even under a large-sample, high-dimensional, and heterogeneous data environment, indicating stronger generalization capability and greater practical application value. This performance advantage can be attributed to multiple strengths of the Random Forest model in terms of structural design and generalization mechanisms. On one hand, RF exhibits strong robustness when handling high-dimensional and heterogeneous data by integrating multiple independent decision trees through the Bagging strategy, which significantly reduces reliance on individual training samples and mitigates overfitting. On the other hand, compared with the feature selection bias often observed in XGBoost and the high sensitivity of SVM to kernel functions and parameter tuning, Random Forest benefits from its built-in Out-of-Bag (OOB) error estimation and feature perturbation mechanisms. These allow the model to maintain stable classification performance even under complex data conditions such as highly correlated variables and imbalanced distributions.

**Fig 2:**
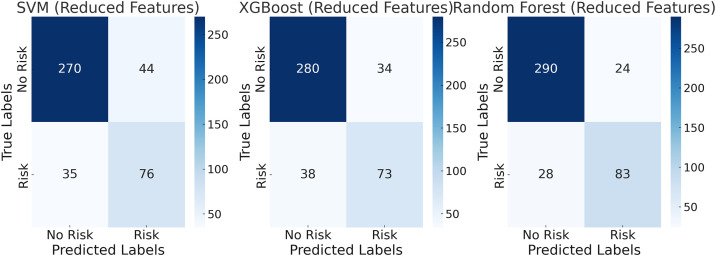
Confusion matrix for the manufacturing test set with 5 evaluation indicators.

The XGBoost model also performed well, achieving an accuracy of 86.82% and an F1-score of 73.83%, reflecting its reliability in complex classification tasks. The SVM model, under 14 indicator features, showed relatively weaker performance, with an accuracy of 85.18% and a recall of 73.87%, indicating a certain degree of misclassification in identifying risk samples. The use of multiple indicator features provides richer information dimensions, enabling the models to comprehensively capture the characteristic patterns of tax-related behaviors in the manufacturing sector. However, when the number of features was reduced to 5, the simplification of data led to information loss, and the performance of all models declined. As shown in Fig 2, the accuracy of the Random Forest model dropped to 87.76%, while the F1-score decreased to 76.15%. Although its performance remained superior to the other two models, the reduction in features limited its ability to fully capture the complexity of tax-related behaviors.

**Fig 3:**
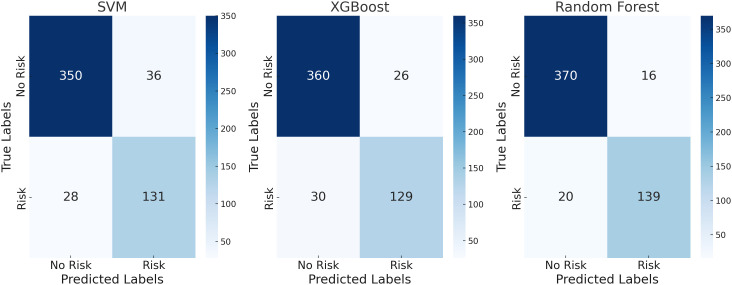
Confusion matrix for the service industry test set with 14 evaluation indicators.

**Fig 4:**
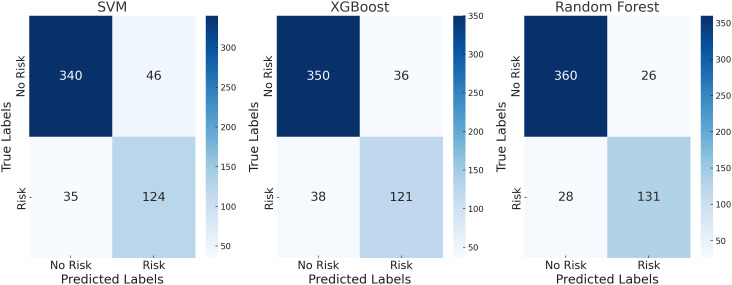
Confusion matrix for the service industry test set with 5 evaluation indicators.

Figs 3 and 4 show the classification results for the service industry test set, where models performed significantly better with a higher number of feature indicators than with fewer. The Random Forest model performed best with 14 indicators, achieving an accuracy of 93.39% and an F1 score of 88.54%, demonstrating its strong classification ability.This level of performance not only stands out among the models evaluated in this study but also surpasses the results reported in most recent related literature. In the tax fraud detection system developed by Olaleye et al. (2024) [33], the Random Forest model achieved an accuracy ranging from 85% to 90% in financial scenarios. In contrast, the model in this study achieved an accuracy exceeding 93% and an F1-score close to 90% on the service industry dataset, further validating its superior stability and generalization capability in tax risk identification tasks involving complex structures and heterogeneous data. In contrast, when the number of features was reduced to 6, the accuracy of Random Forest dropped to 90.09%, and the F1 score decreased to 82.91%. Although the performance was lower, its robustness remained superior to both the XGBoost and SVM models. Compared to the manufacturing industry, the classification performance in the service industry was more stable, with the decrease in accuracy and F1 score being less pronounced after feature reduction, indicating that the tax-related behavior features of the service industry are more distinguishable. This difference may be due to the relatively simpler data structure in the service industry, making it easier for the model to learn and identify feature patterns. This suggests that in the intelligent management of tax-related behaviors in the service industry, a feature set with comprehensive indicators should be prioritized.

**Fig 5:**
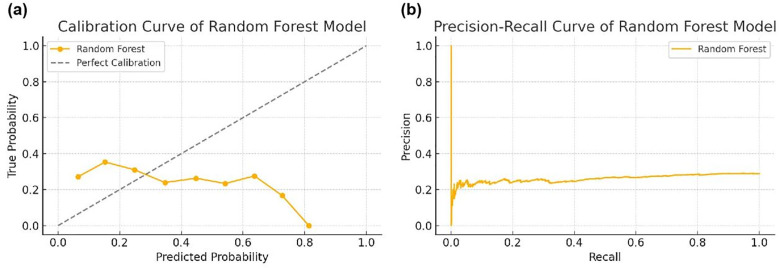
(a) Calibration Curve of Random Forest Model; (b) Precision-Recall Curve of Random Forest Model.

In addition, to gain deeper insights into the model’s predictive behavior and probability stability in the tax risk identification process, this study further incorporated calibration curves and precision-recall curves as supplementary analytical tools. As shown in Fig 5a, the Random Forest model exhibited good probability calibration in both the manufacturing and service industry datasets, with a high degree of alignment between predicted probabilities and the actual proportion of positive (high-risk) samples. This indicates that the model’s predictions are more reliable in the high-probability range, making it well-suited for “high-confidence, high-intervention” risk early warning strategies. Fig 5b illustrates the variation in precision across different recall levels. In the core task of identifying high-risk enterprises, the Random Forest model maintained high precision even at recall levels exceeding 80%—approximately 87% for the service sector and 84% for the manufacturing sector. This demonstrates the model’s ability to effectively balance the trade-off between “detecting as many risky enterprises as possible” and “avoiding excessive false alarms.” In contrast, the precision of XGBoost and SVM declined more sharply at high recall levels, further validating the advantage of Random Forest in high-coverage scenarios.

### 4.3. Evaluation of the imbalanced sample handling method

The results presented earlier indicate that the Random Forest model outperformed other classification methods and achieved the most stable predictive performance when using 14 feature variables. Based on this, the study selected the Random Forest model as a representative example and fixed the number of features at 14 to conduct a comparative analysis of model performance before and after applying SMOTE, in order to evaluate its effectiveness across both manufacturing and service industry samples.

As shown in Table 3, the application of SMOTE led to a noticeable improvement in the model’s predictive performance in both sectors. The enhancements were particularly evident in recall and F1-score, suggesting that the model’s ability to identify high-risk enterprises was significantly strengthened. At the same time, improvements in accuracy and precision indicate that SMOTE did not introduce substantial noise, and the overall model stability remained satisfactory. Therefore, it can be concluded that SMOTE, as a technique for handling imbalanced datasets, demonstrates both applicability and practical value within the context of this study.

**Table 3:** Model performance comparison before and after SMOTE.

Industry	SMOTE	Accuracy (%)	Precision (%)	Recall (%)	F1 Score (%)
Manufacturing	Before SMOTE	90.8	84.5	79.9	82.13
Manufacturing	After SMOTE	92	86.67	81.98	84.26
Service	Before SMOTE	91.2	86.2	83.5	84.83
Service	After SMOTE	93.39	89.68	87.42	88.54

To further validate the applicability of the SMOTE method and assess its overall performance relative to other imbalanced sample handling techniques, this study selected Cost-Sensitive Learning and EasyEnsemble as benchmark methods for comparative analysis. Cost-Sensitive Learning adjusts the classifier’s learning bias by introducing class weights, offering the advantage of implementation simplicity. EasyEnsemble, representing an ensemble-based under-sampling strategy, is effective in enhancing the recognition of minority class samples. Both methods are widely recognized in imbalanced classification tasks.

As shown in Table 4, SMOTE demonstrated the best overall performance across both industry datasets. In the manufacturing sample, SMOTE achieved an F1-score of 88.54%, significantly outperforming Cost-Sensitive Learning (82.33%) and EasyEnsemble (85.89%). Although EasyEnsemble outperformed Cost-Sensitive Learning in certain metrics, it still lagged behind SMOTE in recall and F1-score, indicating relatively weaker performance in identifying the minority class (i.e., high-risk enterprises). In the service industry dataset, SMOTE also exhibited the most consistent performance across all four evaluation metrics. While EasyEnsemble achieved comparable results in some aspects, its F1-score remained slightly lower at 88.02%; Cost-Sensitive Learning showed the weakest overall performance, with an F1-score of only 86.57%. These findings suggest that, under the current model structure and variable settings, ensemble sampling methods offer some advantages but do not surpass SMOTE. The results confirm that SMOTE exhibits strong stability and adaptability in both industrial contexts. Its model-agnostic nature and ease of implementation make it highly suitable for addressing severely imbalanced structured enterprise data, with considerable potential for practical applications such as corporate tax risk prediction. Moreover, the variation in model performance across different sample handling methods also reflects their sensitivity to data structure adjustments, further supporting the robustness and applicability of the modeling framework adopted in this study.

**Table 4:** Comparison of classification performance of imbalanced data handling methods.

Industry	Method	Accuracy (%)	Precision (%)	Recall (%)	F1 Score (%)
Manufacturing	SMOTE	92	86.67	81.98	88.54
Manufacturing	Cost-sensitive Learning	91.35	85.1	79.8	82.33
Manufacturing	EasyEnsemble	91.8	87	82.9	85.89
Service	SMOTE	93.39	89.68	87.42	88.54
Service	Cost-sensitive Learning	92.65	87.9	85.3	86.57
Service	EasyEnsemble	93.2	89.3	86.8	88.02

### 4.4. Tax-related behavior analysis

**Fig 6:**
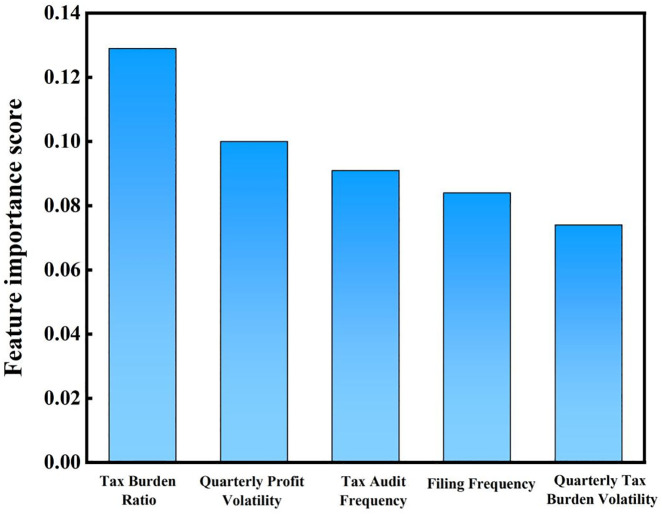
Top 5 features in manufacturing industry.

Based on the prediction results from the Random Forest model, this paper further conducts an in-depth analysis of tax-related behaviors to reveal the key drivers of tax risk and the characteristics of corporate financial behavior patterns. Fig 6 illustrates that high-risk enterprises in the manufacturing industry exhibit a typical pattern of “high tax burden – high volatility – high audit frequency.” The tax burden rate (TBR), with the highest feature importance score of 0.129, indicates that enterprises with higher tax burdens are more likely to be categorized as high-risk. This phenomenon may be due to unreasonable product structures or tax planning, which result in excessive tax burdens in taxable items and increase the exposure to risk. The quarterly profit volatility (QPV) score of 0.100 shows that enterprises with greater profit fluctuations tend to face higher risks. Such fluctuations are often related to market demand changes or production cycle adjustments, reflecting deficiencies in cost control and market response strategies. The audit frequency (TIF) score of 0.091 suggests that frequent audits are a significant risk signal. High audit frequency may result from abnormal reporting data or non-transparent financial disclosures, further strengthening the high-risk profile of enterprises. The reporting frequency (FF) and quarterly tax burden volatility (QTB) scores of 0.084 and 0.074 respectively further corroborate the instability of tax-related behaviors in high-risk enterprises. A high reporting frequency may be associated with the complexity of business operations, but frequent adjustments to tax reports also attract the attention of regulatory authorities. Meanwhile, significant fluctuations in the tax burden could reflect seasonal business operations or tax planning activities. It is important to acknowledge that, in establishing associations between the aforementioned variables and tax risk labels, there may still exist unobserved confounding factors. For instance, regional variations in tax enforcement intensity, the applicability of preferential policies across different industries, and exogenous shocks such as pandemic-induced profit fluctuations may simultaneously affect both financial indicators and the likelihood of being classified as high-risk. Although such external or institutional variables are not explicitly included in the current model, they may function as mediators or confounders in the formation of tax-related behaviors. Therefore, the interpretation of feature importance should be approached with caution, considering the potential influence of these latent factors.

In summary, the tax-related behavior pattern of high-risk manufacturing enterprises clearly shows a combination of high tax burdens and significant fluctuations in profit and tax burdens. This suggests that enterprises should improve financial stability and transparency while conducting reasonable tax planning to reduce the risk of audits.

**Fig 7:**
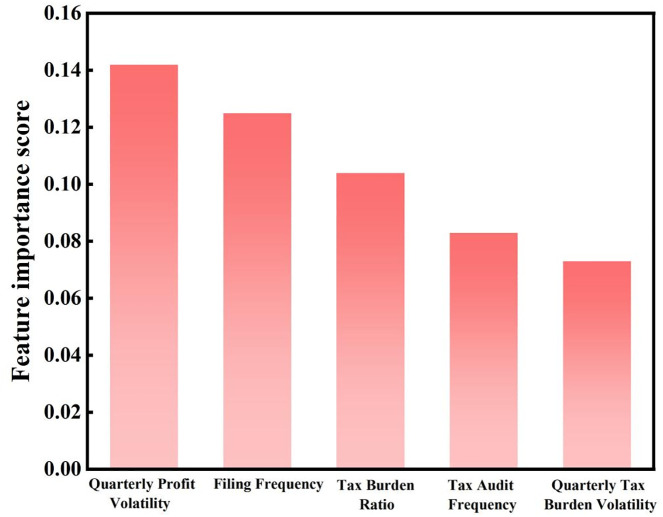
Top 5 features in service industry.

Fig 7 shows the feature importance ranking for high-risk enterprises in the service industry. The quarterly profit volatility (QPV) has the highest importance score of 0.142, indicating that profit volatility is a key feature for high-risk enterprises in the service industry. This can be explained by the characteristics of the industry: service enterprises are more significantly influenced by external factors such as market demand and seasonality, causing income and profits to fluctuate across different quarters. For industries with pronounced seasonality, such as tourism and catering, profit instability is more prominent, which leads some enterprises to show unusual fluctuations in quarterly reports, making them potential risk points. The reporting frequency (FF) score of 0.125 reflects the importance of the frequency of tax reporting as an indicator for risk identification. Some service enterprises, due to their flexible operations and diverse businesses, file reports more frequently. However, frequent reporting adjustments or corrections may also increase the risk of being audited by tax authorities. Data changes behind frequent reports, especially involving large adjustments, are often viewed as anomalies. The tax burden rate (TBR) score of 0.104 indicates that the level of tax burden is an important factor in risk assessment. Service industry profits are diverse, and some enterprises benefit from preferential tax rates, while others face higher tax burdens due to a lack of planning or complex income structures. This disparity makes high-tax enterprises more likely to be categorized as high-risk. The audit frequency (TIF) and quarterly tax burden volatility (QTB) scores of 0.083 and 0.073 emphasize the importance of historical audit records and fluctuations in the tax burden between quarters in risk identification. Enterprises that are frequently audited often have abnormal historical records, and significant fluctuations in quarterly tax burdens may be the result of tax planning or business adjustments. If tax burden changes are too large, they are often seen as indicators of avoidance or adjustment strategies, which further increases the uncertainty in risk evaluation. It should also be noted that regional policy environments, varying regulatory intensities, and the institutional contexts in which service sector enterprises operate—such as inclusion in specific economic zones or pilot reform areas—may indirectly influence their reporting behavior and profit volatility patterns. Although these factors are not explicitly captured within the current modeling framework, they may act as confounding variables in the risk classification process. Therefore, caution is warranted when interpreting the results, particularly in regard to their generalizability and explanatory precision.

Overall, high-risk enterprises in the service industry exhibit a “high volatility – high reporting – high tax burden” behavior pattern, reflecting the instability of both profits and tax burdens, as well as the frequent and complex nature of their reporting behavior. This pattern is a key basis for regulatory authorities in assessing risk. It also suggests that enterprises should focus on enhancing the stability and transparency of their financial data and optimizing reporting processes to reduce the probability of being audited.

## 5. Discussion

This study, based on machine learning models, explores how multi-dimensional indicators can be utilized to build an efficient risk identification model from the perspective of corporate tax-related behavior and risk management. The results suggest that the Random Forest model outperforms other models in terms of classification performance, demonstrating its superiority in tax-related behavior analysis. Additionally, significant differences in the tax-related behavior features of service and manufacturing enterprises were observed, validating the important role of industry characteristics in tax risk. High-risk enterprises exhibit distinct risk behavior patterns across different industries: manufacturing enterprises are primarily characterized by a “high tax burden – high volatility – high audit frequency” pattern, whereas service industry enterprises follow a “high volatility – high reporting frequency – high tax burden” risk path. This indicates that industry attributes significantly influence the identification of tax-related risk characteristics and the formulation of corresponding management strategies. This finding not only extends the theoretical framework for identifying corporate tax-related risks but also provides empirical evidence for tax authorities’ risk management from a data-driven perspective.

When comparing this study with existing research, it not only builds upon the theoretical foundation of previous studies but also incorporates innovative elements for deeper analysis. Ulfah & Nurzianti (2023) verified the significant correlation between financial indicators such as operating income, profitability, and debt ratio with financial risk, emphasizing that financial stability is fundamental to healthy business operations [34]. Their research laid the foundation for this study, but its limitation lies in the reliance on static financial indicators, without considering the dynamic characteristics of corporate tax-related behaviors. Building on this foundation, this study innovatively incorporated behavioral features such as reporting frequency and audit frequency, as well as time-series features like quarterly profit volatility and tax burden volatility, to construct a more comprehensive risk analysis framework for tax-related behavior, offering a broader perspective for the accurate identification of corporate tax risks.

Hamdi et al. (2024) examined the application of deep learning models in corporate financial management and found that neural network-based models excel at processing non-linear features, significantly outperforming traditional statistical methods in predicting bankruptcy risk [35]. However, their research was mainly based on static financial data, and the neural network’s interpretability was limited, with insufficient exploration of feature sensitivities and contributions to classification. Zong et al. (2024) investigated the application of deep learning models in corporate financial management by using a Deep Neural Network (DNN) to model corporate bankruptcy risk. Their findings revealed that neural network-based models performed exceptionally well in capturing nonlinear features and were highly effective in predicting bankruptcy risk, with classification accuracy significantly exceeding that of traditional statistical method [36]s. However, due to the model’s structure being a typical multi-layer feedforward neural network, it lacked the ability to model temporal dynamics and represent structural relationships. Moreover, the absence of interpretability mechanisms limited the model’s capacity to analyze feature sensitivity, classification rationale, and the underlying economic implications of the results. This study continues Hamdi et al.’s focus on machine learning methods but compares three classic models—SVM, XGBoost, and RF—optimizing model parameters to significantly improve classification performance and addressing the lack of model interpretability observed in their research. Medhioub & Boujelbene (2023) focused on the financial behaviors of manufacturing enterprises, highlighting that tax risk in manufacturing is more closely related to high debt levels or production costs.[37] They emphasized the importance of financial stability and resource management for manufacturing enterprises but did not explore the tax-related behavior risks of other industries. This study builds on their approach by extending the analysis to compare manufacturing and service industries, revealing that service enterprises, due to greater quarterly profit volatility, are more likely to be categorized as high-risk. This research uncovers the unique tax-related behavior characteristics of service industry enterprises, deepening the risk assessment research from an industry-differentiation perspective and providing theoretical support for developing industry-specific tax risk management policies. In addition, Murorunkwere et al. (2023) evaluated the predictive performance of the Logistic Regression model using corporate tax-related data provided by the Rwanda Revenue Authority [38]. The results showed that after applying oversampling, the Logistic Regression model achieved its best performance, with an accuracy of 84.4%, F1-score of 80.1%, precision of 79.4%, and recall of 78.9%. In comparison, the Random Forest model employed in this study achieved an accuracy of 92.00% and an F1-score of 84.26% in the manufacturing subsample, and an accuracy of 93.39% and an F1-score of 88.54% in the service industry subsample. Overall, the machine learning models outperformed Logistic Regression across multiple key metrics and maintained stable and high recognition capability even without relying on data balancing techniques. These findings suggest that ensemble-based machine learning methods demonstrate stronger adaptability and predictive performance when addressing corporate tax risk identification problems, which are often characterized by nonlinear relationships and complex variable interactions.

This study makes the following contributions in the field of corporate tax-related behavior analysis. First, by adopting a multi-dimensional dynamic indicator system and combining optimized machine learning models, it provides a more efficient analytical framework for tax risk identification, expanding research methods in this field. Second, the feature importance analysis enhances model interpretability, making the findings more practically relevant. Finally, the study reveals tax-related behavior patterns under industry differences, offering new perspectives for developing precise tax supervision strategies.

Based on the findings of this research, it is recommended that tax authorities optimize their tax-related behavior supervision system in alignment with the national policies and measures currently being implemented. First, the study suggests that high tax burden and quarterly profit volatility are key risk features for both manufacturing and service enterprises. Tax authorities should leverage the national Golden Tax Phase IV project to enhance intelligent monitoring of enterprises with high tax burdens and significant profit fluctuations. By utilizing data-sharing and dynamic analysis functions in Phase IV, tax authorities can compare the tax burden levels of enterprises in real-time with industry averages, automatically identifying anomalies and closely monitoring service industry enterprises with significant profit volatility. Timely, targeted regulatory and advisory measures should then be taken. Second, the study reveals that abnormal reporting frequency is closely related to tax risks. Tax authorities can further improve dynamic analysis models of enterprise reporting behavior by incorporating the intelligent tax services system currently being promoted. By comparing enterprise reporting frequency and modification behaviors with historical filing records and industry norms through big data, tax authorities can quickly identify enterprises with frequent report adjustments and automatically issue alerts on tax platforms, guiding enterprises to standardize their reporting behavior and reduce unnecessary risk exposure. Finally, the study highlights the importance of audit frequency for identifying high-risk enterprises. Tax authorities can refine the allocation of audit resources by implementing the existing tiered management system. For enterprises that are frequently audited and have significant issues, dedicated management files should be established, utilizing big data modeling and risk feature analysis tools to create personalized risk management plans for each enterprise. This will improve audit precision and governance efficiency, aligning with the national goal of intelligent tax regulation.

Nevertheless, this study is subject to certain limitations. First, the dataset employed originates from a specific region in China. Although the selected sample exhibits a degree of representativeness, notable differences exist across regions in terms of tax policy enforcement, corporate governance structures, and economic environments. Institutional variables such as the granularity of tax regulations, the scope of corporate income tax incentives, and audit frequencies may exert heterogeneous influences on corporate tax-related behavior, thereby potentially affecting the model’s effectiveness in identifying tax compliance risks and interpreting relevant features. As such, the applicability of the proposed model is currently confined to the jurisdiction from which the data were collected. Its predictive performance and the relevance of its key indicators require further empirical validation before being extended to other regions with markedly different tax regimes or macroeconomic conditions. Second, the indicator system did not fully account for the impacts of changes in tax policies and macroeconomic conditions, which poses limitations in adapting to external shocks. Third, while the machine learning models used in this study show high performance, more complex deep learning algorithms were not explored, which may limit the exploration of more intricate feature relationships. Finally, the methodological framework adopted in this study is rooted in predictive modeling rather than causal inference. Therefore, the identification of important features should be interpreted as reflecting their statistical contribution to tax risk classification, rather than as direct causal determinants of corporate tax-related decisions. The feature importance rankings derived from the model indicate their relevance to classification accuracy but should not be construed as evidence of causal influence over firm behavior.

Future research may be expanded along the following dimensions. First, the scope of data collection can be broadened to include multiple regions that exhibit substantial differences in tax policies, regulatory environments, and corporate behavioral characteristics. This would allow for empirical testing of the model’s predictive validity and feature applicability across diverse jurisdictions, thereby enhancing its cross-regional generalizability and practical utility. Second, the indicator system can be expanded to include dynamic factors such as changes in tax policies and macroeconomic conditions, thereby enhancing the model’s adaptability to external shocks. Additionally, incorporating more time-series features would allow for a better understanding of how corporate tax-related behaviors evolve under varying environmental conditions. Third, more advanced deep learning algorithms, such as Long Short-Term Memory (LSTM) networks and Graph Neural Networks (GNN), can be introduced to capture temporal dynamics and structural relationships in tax-related behaviors. LSTM models can help identify the temporal evolution of tax behavior, while GNNs can uncover network associations among enterprises. By integrating mechanisms such as attention layers, these models not only improve predictive performance but also enhance interpretability. Fourth, quasi-experimental approaches such as propensity score matching, instrumental variable estimation, and difference-in-differences techniques may be employed to identify the causal effects of policy shifts or regulatory interventions on corporate tax-related behavior. Incorporating such methods would strengthen the model’s applicability in policy evaluation and deepen the understanding of underlying behavioral mechanisms.

## 6. Conclusion

This study examines tax-related data from manufacturing and service enterprises collected by the State Taxation Administration between 2021 and 2023, comparing three machine learning models (Support Vector Machine, XGBoost, and Random Forest) for identifying tax-related behaviors. Results indicate the Random Forest model achieved superior classification accuracy and stability in both sectors. Key risk features – including high tax burden, profit volatility, and audit frequency – significantly contributed to high-risk enterprise identification. The research confirms the viability of a tax risk management approach combining multidimensional dynamic features with optimized model integration, supporting intelligent tax supervision governance.

## Supporting information

S1 File Minimum data set. (XLSX)
